# A novel hamster model of SARS-CoV-2 respiratory infection using a pseudotyped virus

**DOI:** 10.1038/s41598-022-15258-8

**Published:** 2022-07-01

**Authors:** Hiroshi Yamada, So-Ichiro Sasaki, Hideki Tani, Mayu Somekawa, Hitoshi Kawasuji, Yumiko Saga, Yoshihiro Yoshida, Yoshihiro Yamamoto, Yoshihiro Hayakawa, Yoshitomo Morinaga

**Affiliations:** 1 Department of Microbiology, Graduate School of Medicine and Pharmaceutical Sciences, University of Toyama, 2630 Sugitani, Toyama, 930-0194 Japan; 2 Section of Host Defences, Institute of Natural Medicine, University of Toyama, Toyama, Japan; 3 Department of Virology, Toyama Institute of Health, Toyama, Japan; 4 Department of Clinical Infectious Diseases, Toyama University Graduate School of Medicine and Pharmaceutical Sciences, Toyama, Japan

**Keywords:** Virology, Infectious diseases

## Abstract

Severe acute respiratory syndrome coronavirus 2 (SARS-CoV-2) is a biosafety level (BSL)-3 pathogen; therefore, its research environment is limited. Pseudotyped viruses that mimic the infection of SARS-CoV-2 have been widely used for in vitro evaluation because they are available in BSL-2 containment laboratories. However, in vivo application is inadequate. Therefore, animal models instigated with animal BSL-2 will provide opportunities for in vivo evaluation. Hamsters (6–10-week-old males) were intratracheally inoculated with luciferase-expressing vesicular stomatitis virus (VSV)-based SARS-CoV-2 pseudotyped virus. The lungs were harvested 24–72 h after inoculation and luminescence was measured using an in vivo imaging system. Lung luminescence after inoculation with the SARS-CoV-2 pseudotyped virus increased in a dose-dependent manner and peaked at 48 h. The VSV-G (envelope G) pseudotyped virus also induced luminescence; however, a 100-fold concentration was required to reach a level similar to that of the SARS-CoV-2 pseudotyped virus. The SARS-CoV-2 pseudotyped virus is applicable to SARS-CoV-2 respiratory infections in a hamster model. Because of the single-round infectious virus, the model can be used to study the steps from viral binding to entry, which will be useful for future research on SARS-CoV-2 entry without using live SARS-CoV-2 or transgenic animals.

## Introduction

Severe acute respiratory syndrome coronavirus 2 (SARS-CoV-2) causes coronavirus disease 2019 (COVID-19) and has seriously affected our health and social activities. There is an urgent need to elucidate the pathophysiology of COVID-19 and to develop treatment and prophylactic approaches.

SARS-CoV-2 is classified as a biosafety level 3 (BSL-3) pathogen and requires a limited laboratory environment and strict control by skilled researchers^1^. Research using live viruses has the advantage of evaluating disease pathogenicity more directly; however, there are some limitations in laboratories and efforts. Therefore, pseudotyped viruses, which are single-round infectious virus particles with an envelope protein originating from a different virus^2^, have been widely used in COVID-19-related research because they can be used in BSL-2 containment laboratories. As an alternative to live SARS-CoV-2, we previously developed a pseudotyped virus, vesicular stomatitis virus (VSV), expressing luciferase, using the truncated spike (S) proteins of SARS-CoV-2^3^. The luminescence observed after treatment with luciferin reflected viral infection in cells and was highly infectious to humans (Huh7 and 293 T), hamsters (CHO), and monkeys (Vero)^3^. Pseudotyped viral systems have been widely used for in vitro evaluations, such as neutralization activity^3–5^; however, whether they can also be used in animal models remains unknown.

SARS-CoV-2 uses angiotensin-converting enzyme 2 (ACE2) on the cell surface to bind to and enter cells. The SARS-CoV-2 S protein does not effectively bind to mouse ACE2^6^; therefore, sensitivity to infection is species-dependent. Thus, in vivo live virus infection models have been reported in SARS-CoV-2-sensitive animals, such as Syrian hamsters, transgenic mice expressing human ACE2, and ferrets^7^.

Animal models that can be instigated with Animal Biosafety Levels 2 (ABSL-2) will increase opportunities for in vivo evaluation. Therefore, in this study, a hamster model of SARS-CoV-2 respiratory infection using a VSV-based pseudotyped virus was established. Infectivity can be evaluated by in vivo imaging or quantification of the VSV N gene expression. Because all procedures can be performed in BSL/ABSL-2 facilities, it expands the possibility of various ideas related to COVID-19 that could not be evaluated due to BSL3 limitations.

## Materials and methods

### Generation of pseudotyped viruses

Pseudotyped VSVs bearing SARS-CoV-2 S proteins were generated, as previously described^3^. The expression plasmid for the truncated S protein of SARS-CoV-2 and pCAG-SARS-CoV-2 S (hCoV-19/Japan/TY/WK-521/2020) was provided by Dr. Shuetsu Fukushi of the National Institute of Infectious Diseases, Japan. The S cDNA of SARS-CoV-2 with a 19 amino acid (aa) truncation at the C-terminus was cloned into the pCAGGS expression vector. Next, 293 T cells were grown to 80% confluence on collagen-coated tissue culture plates and transfected with each expression vector. After 24 h of incubation, cells transfected with each plasmid were infected with G-complemented (*G) VSV∆G/Luc (*G-VSV∆G/Luc) at a multiplicity of infection (MOI) of 0.5 per cell. After 24 h of incubation, the culture supernatants containing pseudotyped VSVs were centrifuged and stored at −80 °C until ready for use. Pseudotyped VSVs bearing envelopes (G) (VSV-G) were also generated. The pseudotyped VSVs were stored at −80 °C until subsequent use. Finally, VeroE6/TMPRSS2 cells (JCRB1819) were infected with the viral solution, the fluorescence level was measured, and the concentration of the solution was adjusted to approximately 5 × 10^4^–5 × 10^5^ RLU/μL for in vivo experiments (onefold concentration).

### Animals

All experiments and methods were performed in accordance with the relevant guidelines and regulations. All experimental protocols used in this study were approved by the Animal Care and Use Committee of the University of Toyama (Protocol Number: A2020MED-18). Experiments and analyses were performed according to the ARRIVE guidelines.

### Hamster models

Six-to-10-week-old male hamsters were purchased from Japan SLC Inc. (Shizuoka, Japan). All animals were housed in a pathogen-free environment in the Division of Animal Resources and Development at the University of Toyama.

**Figure 1:**
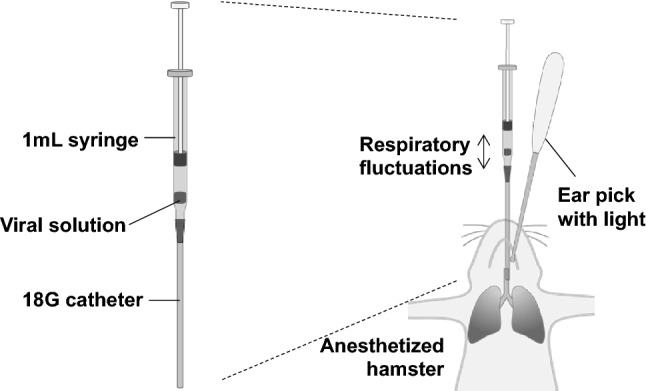
Schematic illustration of the inoculation of virus solution into the hamster airway. A viral solution (100 μL/hamster) with a small amount of air was prepared in a 1 mL syringe attached to an 18G catheter. After observing the glottis of the anesthetized hamster with an ear pick with light, the syringe set containing the viral solution was inserted through the oral cavity and advanced through the vocal cords. The respiratory fluctuations of the viral solution indicated that the tip of the catheter was in the trachea.

After titration of the viral solution, 100 μL (7.1 × 10^5^–10^6^ RLU/hamster for SARS-CoV-2 pseudotyped virus [SARS-CoV-2pv] and 7.1 × 10^7^–10^8^ RLU/hamster for VSV-G pseudotyped virus [VSV-Gpv]) were directly inoculated into the trachea, as previously described^8^. Briefly, after anesthesia with isoflurane or a mixture of medetomidine (0.15 mg/kg), midazolam (2 mg/kg), and butorphanol (2.5 mg/kg), viral solutions were administered through the trachea using an 18 G (65 mm long) catheter (TOP Co., Tokyo, Japan) with a 1 mL syringe under the assistance of an ear pick with light (Fig. 1). Immediately after confirming that the solution in the syringe showed respiratory fluctuations, the viral solution was administered to the lower respiratory tract.

### Bioluminescent imaging

The hamsters were sacrificed using isoflurane and cervical dislocation 24 h after infection. The lungs were harvested and washed with phosphate-buffered saline (Nakarai Tesque, Kyoto, Japan). The lungs were incubated with 1 mg/mL of D-luciferin (Promega, Madison, WI, USA) for 5 min, and luminescence was measured using an in vivo imaging system (IVIS Lumina II, Perkin Elmer, MA, USA). Analyses were performed using Living Image 4.2 software (Caliper Life Science) to measure the light emitted from the infection sites. Luminescence from the front and back of the lungs was measured and the sum was calculated. All values are expressed as photons per second per cm^2^ per steradian (p/sec/cm^2^/sr) for each hamster.

### Quantification of VSV N expression

To evaluate viral infection, VSV N gene expression in the lungs was measured by quantitative polymerase chain reaction^9^. The lungs were homogenized in a tube containing glass beads and 500 μL of pre-chilled phosphate-buffered saline using BeadMill 24 (Thermo Fisher Scientific, MA) at a speed of 6 m/s for 60 s. Each 20 μL of homogenized lung solution was immediately mixed with 500 μL of Isogen (Nippon Gene, Toyama, Japan) and stored at −80 °C until subsequent use. Total RNA was extracted using a QIAamp Viral RNA Mini Kit (Qiagen, Hilden, Germany), according to the manufacturer’s instructions. Total RNA was reverse-transcribed into cDNA and then amplified using Thunderbird SYBR qPCR/RT Set III (TOYOBO Co.., Tsuruga, Japan). VSV N expression was normalized to that of γ-actin^10^.

### Statistical analysis

The luminescence data are expressed as the mean ± standard error of the mean (SEM). Differences between low and high SARS-CoV-2pv concentrations were examined for statistical significance using an unpaired Student’s t-test with GraphPad Prism version 8.4.3 (GraphPad Software, CA, USA). Because high values for higher SARS-CoV-2pv concentrations were expected, a one-tailed P value of < 0.05 denoted the presence of a statistically significant difference.

## Results

### Intratracheal inoculation of SARS-CoV-2 pseudotyped virus demonstrated signs of lung infection

To develop a SARS-CoV-2 pseudotyped viral infection model, we first inoculated VSV-Gpv or SARS-CoV-2pv intranasally; however, no luminescence was observed by bioluminescent imaging (data not shown). Thus, the viral solutions were inoculated intratracheally (Fig. 1). Inoculation with VSV-Gpv or SARS-CoV-2pv induced no clinical signs until 24 h after inoculation.

**Figure 2:**
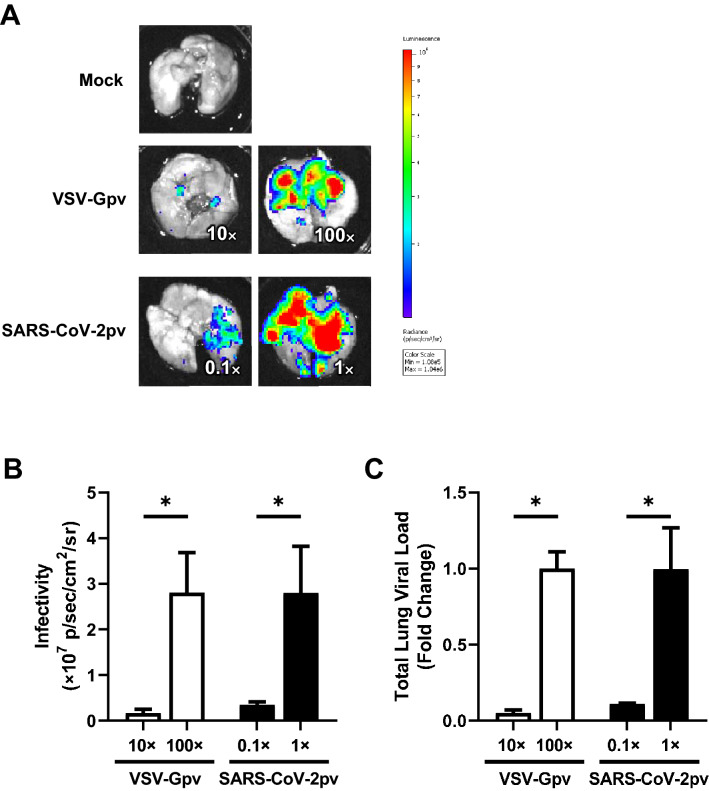
Dose-dependent infectivity after inoculation of the pseudotyped virus. (**A**) Representative lung imaging after inoculation of mock (top), VSV-Gpv (middle), and SARS-CoV-2pv (bottom). The numbers indicate the concentration ratio from the original viral solution (7.1 × 10^6^ RLU/hamster). (**B**) Infectivity after inoculation of VSV-Gpv (n = 3) and SARS-CoV-2pv (n = 3). Data are expressed as the mean of sum luminescence of the front and back. (**C**) Viral loads in lungs evaluating VSV N expression. Data are expressed as the fold change from the higher concentration (VSV-Gpv; n = 2, SARS-CoV-2pv; n = 3). All experiments were performed 24 h after inoculation. Error bars are the SEM. * indicates p < 0.05.

For VSV-Gpv, no luminescence was detected in the lungs inoculated with 7.1 × 10^6^ RLU/hamster (onefold concentration; data not shown). Thus, higher concentrations of VSV-Gpv (10- and 100-fold) were inoculated (Fig. 2A). The luminescence values of the 10- and 100-fold concentrations were 0.16 ± 0.08 × 10^7^ and 2.8 ± 0.88 × 10^7^ p/sec/cm^2^/sr, respectively (p < 0.05, n = 3 each) (Fig. 2B).

In contrast, luminescence was clearly observed when 7.1 × 10^6^ RLU SARS-CoV-2pv was inoculated (Fig. 2A). The mean ± SEM of the luminescence values after inoculation of 7.1 × 10^5^ RLU/hamster (0.1-fold concentration) and 7.1 × 10^6^ RLU/hamster (onefold concentration) were 0.35 ± 0.11 × 10^7^ and 2.80 ± 1.8 × 10^7^ p/sec/cm^2^/sr, respectively (p < 0.05, n = 3 each) (Fig. 2B). The viral loads in the lungs increased in a dose-dependent manner in the VSV-Gpv and SARS-CoV-2pv inoculated hamsters (Fig. 2C).

Thus, luminescence values and viral loads reflected the amount of VSVGpv inoculated.

### Signals of SARS-CoV-2 pseudotyped virus lung infection peaked at 48 h after inoculation

**Figure 3:**
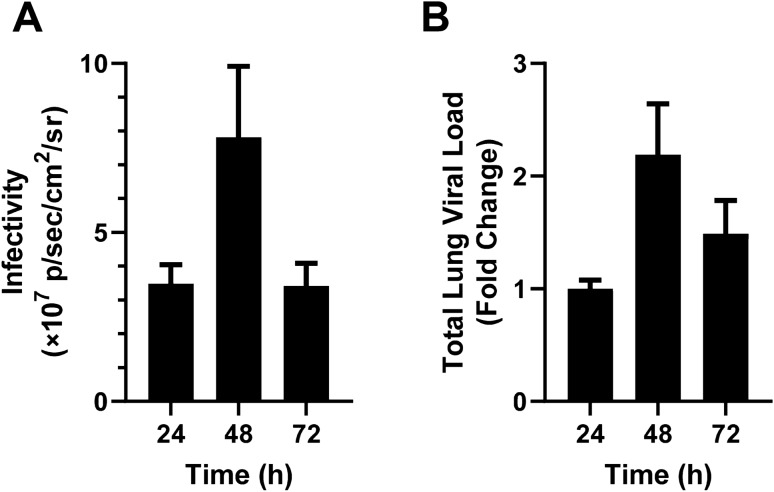
Time course of infectivity after inoculation of the pseudotyped virus. (**A**) Time course of luminescence levels after inoculation of SARS-CoV-2pv (n = 3, each). Data are expressed as the mean of sum luminescence of the front and back. (**B**) Time course of viral loads in lungs (n = 3, each). Data are expressed as the fold change from the higher concentration.

To confirm the time course of the model, luminescence levels and viral loads were measured until 72 h after inoculation. The luminescence values peaked 48 h after inoculation and then decreased to the same level as that 24 h after inoculation (Fig. 3A). The viral loads in the lungs also peaked at 48 h after inoculation and then slightly decreased at 72 h (Fig. 3B).

These results indicate that VSV-Gpv infection persisted for up to 48 h with increasing signals.

## Discussion

Our results demonstrated that VSV-based SARS-CoV-2pv was applicable to SARS-CoV-2 respiratory infection in the hamster model. This method could be used as an alternative model for experiments in an ABSL-3 facility or to avoid handling highly infectious viruses, and it expands the possibility of analysis by using equipment that cannot be used with BSL-3/ABSL-3.

A lower respiratory infection model using pseudotyped SARS-CoV-2 has not been reported to date. Although a mouse model using lentivirus-based SARS-CoV-2pv following intranasal administration of human ACE2 has been reported^11^, the virus did not infect the lower respiratory tract and remained around the nose. Generally, nasal inoculation is a convenient route for inducing lower respiratory tract infections. However, in our preliminary experiments, sufficient luminescence was not observed by in vivo imaging after nasal inoculation with the pseudotyped virus (data not shown). Therefore, it is essential to approach the lower respiratory tract directly. A tracheostomy approach from the anterior neck is a possible inoculation route for pathogens into the lower respiratory tract^12^; however, our intratracheal approach is less invasive and complicated. Confirmation of respiratory fluid fluctuations provided good reproducibility of the models. The time required for inoculation was approximately 1–3 min per hamster.

Our model can be used for research on the viral binding steps. For example, it would be useful in research on the treatment and prevention of viral binding and entry. However, this model is unsuitable for assessing advanced status, such as severe conditions and extrapulmonary infections, because of the single-round infectious virus. Because the viral solution was sprayed blindly, the infected lesion might have been one-sided, and it was better to evaluate both the front and back sides. The imaging findings were consistent with VSV N expression, suggesting that in vivo imaging is a suitable assay for evaluating the viral load. In the present study, despite inoculation with the same infectivity VSV-Gpv in vitro, an approximately 100-fold concentration was required to show relative luminescence in vivo. This finding could be a result of the expression ratios of the low-density lipoprotein receptor used by VSV^13^, and the ACE2 receptor used by SARS-CoV-2 differing in vitro and in vivo. However, VSV-Gpv could be used as a control in intervention studies by inoculating with a 100-fold dose if required.

The model can be useful as an assessment model for the evaluation of treatment interventions, such as antiviral drugs or antibodies, for at least 24 to 48 h. The appropriate time for the evaluation depends on the intervention; however, it could be easy to obtain data with good reproducibility until 48 h. On the other hand, because the natural decline in luminescence levels and viral loads was observed at 72 h, it could be inappropriate for treatment evaluation.

In conclusion, we successfully established a hamster model of SARS-CoV-2 respiratory infection by using a VSV-based pseudotyped virus. This model can be used to evaluate treatment and preventive potential without using highly infective pathogens and transgenic animals. As it is not limited to ABSL-3 containment, further studies can evaluate different ideas, which will be useful for future COVID-19 studies.

## Data Availability

There is no additional data.
