# Author Correction: A novel hamster model of SARS-CoV-2 respiratory infection using a pseudotyped virus

**DOI:** 10.1038/s41598-022-25678-1

**Published:** 2022-12-07

**Authors:** Hiroshi Yamada, So-Ichiro Sasaki, Hideki Tani, Mayu Somekawa, Hitoshi Kawasuji, Yumiko Saga, Yoshihiro Yoshida, Yoshihiro Yamamoto, Yoshihiro Hayakawa, Yoshitomo Morinaga

**Affiliations:** 1 Department of Microbiology, Graduate School of Medicine and Pharmaceutical Sciences, University of Toyama, 2630 Sugitani, Toyama, 930-0194 Japan; 2 Section of Host Defences, Institute of Natural Medicine, University of Toyama, Toyama, Japan; 3 Department of Virology, Toyama Institute of Health, Toyama, Japan; 4 Department of Clinical Infectious Diseases, Toyama University Graduate School of Medicine and Pharmaceutical Sciences, Toyama, Japan

Correction to: *Scientific Reports* https://doi.org/10.1038/s41598-022-15258-8, published online 01 July 2022

The Funding section in the original version of this Article was incomplete.

“This study was supported by the Research Program on Emerging and Re-emerging Infectious Diseases from AMED Grant No. JP20he0622035 (HT, YYa, and YM), and the Research Funding Grant by the President of the University of Toyama (YYa, and YM).”

now reads:

“This study was supported by the Research Program on Emerging and Re-emerging Infectious Diseases from AMED Grant No. JP20he0622035 (HT, YYa, and YM), the Research Funding Grant by the President of the University of Toyama (YYa, and YM), and the Toyama Pharmaceutical Valley Development Consortium (YM)”.

The original Article has been corrected.

